# Statin consumption as a risk factor for developing colorectal cancer: a retrospective case study

**DOI:** 10.1186/s12957-017-1287-0

**Published:** 2017-12-16

**Authors:** David Renman, Erik Lundberg, Ulf Gunnarsson, Karin Strigård

**Affiliations:** Institution of Surgery and Perioperative Science, Umeå University, 90185 Umeå, Sweden

**Keywords:** Colorectal cancer, Statin, Risk factor, Reactive oxygen species, Mitochondrial DNA damage, Diabetes mellitus

## Abstract

**Background:**

Statins are the backbone of lipid-lowering therapy and are among the most commonly prescribed drugs in the elderly population in Sweden today. Colorectal cancer is the second most common cancer in men and women, after prostate and breast cancer, respectively, with a median age of 72 years at diagnosis. Statins induce mitochondrial damage leading to accumulation of reactive oxygen species in the cell. Reactive oxygen species can cause mutations in mitochondrial as well as nuclear DNA leading to the development of cancer. Our hypothesis was that statins increase the risk for colorectal cancer.

**Methods:**

A case study was performed on consecutive cases of colorectal cancer diagnosed at Norrlands University Hospital (NUS) in Umeå between 2012 and 2015 (*n* = 325). Patients diagnosed with diabetes mellitus type II (DM II *n* = 65) were excluded in the primary endpoint analysis (occurrence of colorectal cancer). As control, three databases were used to create an age-matched population in order to calculate the proportion of inhabitants using statins in the county of Västerbotten, Sweden. A secondary endpoint was cancer-specific survival among our study group of colorectal cancer patients, including those with DM II, investigating whether there was a difference if the patient was a ‘recent’ statin user or not at the time of diagnosis.

**Results:**

Statin use at the time of colorectal cancer diagnosis in the study group was 23.8%. The corresponding figure in an age-matched population in Västerbotten was 24.6%. Using a one-proportional one-sided *z* test, there was no significant difference between these (23.8%, 95% CI 18.6–29.0%, *p* = 0.601). When comparing groups 20–64 years of age, the difference was greater with recent statin use in 17.8% in the study population and 11.9% in Västerbotten (17.8%, 95% CI 9.0–26.6%, *p* = 0.059). When considering cancer-specific survival, no significant difference in survival was seen when comparing ‘former/never’ statin users as reference category with ‘recent’ users diagnosed with colorectal cancer (HR 1.39, 95% CI 0.89–2.16).

**Conclusions:**

No significant increase in risk for developing colorectal cancer among patients (type II diabetics excluded) medicated with statins was found. We found no correlation between ‘recent’ statin use at the time of diagnosis and cancer-specific survival.

## Background

Statins (hydroxymethylglutaryl-CoA reductase inhibitors) are the third most prescribed drugs in Sweden for long-term use, after beta-blockers and acetylsalicylic acid. In 2008, more than 760,000 Swedish inhabitants were prescribed statins, and in the age group 75–84 years, one in every three citizens was treated with a statin [1]. Elderly patients commonly present with polypharmacy; Swedes over 75 years on average are prescribed more than five drugs per capita [2]. Polypharmacy itself is a risk factor for adverse events due to drugs [2] partly due to known and unknown interactions between drugs.

Colorectal cancer (CRC) is the second most common cancer in Sweden following prostate and breast cancer in men and women, respectively. The risk for CRC increases with age, and nearly 70% of patients with colon cancer are over 65 years at the time of diagnosis [3].

It is well established that statins have cardiovascular protective effects [4]. It has also been shown that statins lower cardiovascular risk even in patients without hyperlipidaemia [5]. It is generally assumed that statins have properties other than lowering lipid levels, called pleiotropic effects. A consequence of these effects has led to the suggestion that statins might play a part in carcinogenesis. Hypotheses concerning the pleiotropic effects of statins are numerous and include pro-apoptotic effects [6], upregulated mevalonate synthesis in extrahepatic tissue, changes in mitochondrial membrane potential [7] and immune system modulation [8]. Statins have been shown to induce oxidative stress in colon cancer cells [9]. Furthermore, statins are mostly eliminated in an unchanged state by the liver into the intestine [10].

Mitochondrial damage is thought to precede degenerative diseases, ageing and cancer [11]. Theories include mitochondrial damage generating reactive oxygen species (ROS) that cause mutations in mitochondrial DNA (mtDNA) and nuclear DNA (nDNA) [11]. ROS also stimulate tumour promotion by driving cellular proliferation [11–13]. MtDNA mutation has been demonstrated in CRC. These mutations are homoplasmic, indicating that they are derived from the tumour line [14]. A recent study indicated that one of statins’ pleiotropic effects is to generate ROS in mitochondria [15]. It is hypothesised that the generation of ROS that can induce apoptosis in carcinoma cell lines [9] can also induce carcinogenesis in healthy cells by promoting ageing of the cell and causing DNA mutations. Opinions differ on whether statins increase [16–18], reduce [19–21] or have no effect on colorectal carcinogenesis [22–31].

Due to the widespread prescription of statins in the elderly Swedish population, it is important to investigate the incidence of possible negative side effects of statins. Increased knowledge about such adverse effects could lead to restrictions in statin prescription and to facilitate direction of the newly suggested screening program for the Swedish population aged between 60 and 74 years old, incorporated from European guidelines [32]. Meta-analyses investigating the relationship between the use of statins and the occurrence of CRC, regardless of conclusions reached, highlight the need for further investigation in this field. RCTs in the field of carcinogenesis are always difficult to carry out due to the length of time it takes to develop cancer. We are thus limited to register- and medical record-based studies in this important field, if we are to deepen our knowledge and, in the future, perhaps modulate indications for statin prescription.

The primary aim of this study was to test the hypothesis that statins increase the risk of developing CRC in patients without diabetes mellitus type II (DM II). Our secondary aim was to investigate if there is any difference in survival between ‘recent’ and ‘former/never’ users of statins in patients (including those with DM II) diagnosed with CRC.

## Methods

This was a pilot retrospective case study where consecutive CRC cases diagnosed at Norrland’s University hospital (NUS) between 2012 and 2015 were included. A search in the medical record database was conducted searching for ICD-10 codes C18-C20. A total of 735 unique patients 2012–2015 had had one or more contacts with the surgical department at NUS, either as an out-patient, as patient admitted to the ward, or as a patient consultation. Of these patients, 397 were excluded since they either had their CRC diagnosed before 2012 or lived in an area that did not come under the NUS catchment area for newly diagnosed CRC. Patients with anal cancer (*n* = 10) and neuroendocrine tumours (*n* = 2) that had been wrongly diagnosed as CRC were excluded from the study. One patient was excluded due to participation in a randomised controlled study where patients were randomised to either statin use or placebo. This left 325 cases of CRC diagnosed at NUS between Jan. 1, 2012, and Dec. 31, 2015, for analysis. Diabetes is a risk factor for CRC [33], and statins are prescribed to almost all diabetic patients in Sweden as primary prevention, often already in early stages of life. Due to this, there is considerable risk of confounding results, and thus, the causality in the results can be altered. For this reason, patients with diabetes mellitus type II (*n* = 65) were excluded from statistical analysis of the primary endpoint.

### Identification of statin use

In order to identify patients who had been prescribed statins, a search in the drug module of the medical record database was conducted. Individuals identified as having had two or more statin prescriptions and at least one of them more than 1 year prior to the cancer diagnosis were defined as ‘has used’ statin users while all the others were defined as ‘never’ users. We also subdivided ‘has used’ users into the categories ‘recent’ user which meant at least one prescription within 3 years prior to diagnosis and ‘former’ user which was defined as having all prescriptions 3 or more years prior to the diagnosis of cancer.

### Database

The study database was managed by Microsoft Access 2016® (Microsoft Office, Redmond, Washington, USA) with data including date at diagnosis; age when diagnosed; date of death; diagnosis (ICD-10: C18, C20); gender; statin use (subdivided into ‘recent’ use and ‘former/never’ use, duration of statin use (short term < 3 years, medium term 3–5 years and long term > 5 years) and type of statin); non-steroidal anti-inflammatory drug (NSAID) use (subdivided into former/recent use); salicylic acid use (subdivided into former/recent use); previous cancer diagnosis; C-reactive protein (CRP) at diagnosis; carcinoembryonic antigen (CEA) at diagnosis; tumour, node, metastasis (TNM) classification; haemoglobin at diagnosis; diabetes (type and medication); blood pressure; heredity for CRC; colitis; height; weight; BMI; smoking habit (divided into current (=within 1 year prior to diagnosis), prior (=ever smoker, but not current), not currently (=documented as ‘non-smoker’ but not clear in medical records whether never or prior smoker) and never smoker); previous cholecystectomy; atherosclerotic disease (ICD-10: I20, I24, I25, I70); myocardial infarction (ICD-10: I21-I23.9); stroke (ICD-10: I61, I63, I64); and alcohol abuse (ICD-10: F10, G31.2, G62.1, G72.1, I42.6, K29.2, K86.0, R78.0, T51, Z72.1). NSAID ever use and salicylic acid ever use were defined as two or more prescriptions and at least one prescription more than 1 year prior to the CRC diagnosis with all the others defined as never/rare users. TNM data was extracted from medical records; from multidisciplinary conferences following any procedure where biopsies from lymphatic nodes had been analysed. For other diagnoses such as atherosclerotic disease, myocardial infarction, stroke and alcohol abuse, we used the ‘patient summary’ in the medical record system and searched for the abovementioned ICD-10 codes.

### Control group

Our primary aim was to compare statin use among CRC cases with statin use in the background population in the county of Västerbotten. The Swedish National Board of Health and Welfare provides a database of prescribed drugs that have been taken out in pharmacies throughout Sweden 2006–2015. These data can further be divided into counties such as Västerbotten [1]. The population register, kept by the Central Bureau of Statistics, was used to calculate the number of Västerbotten inhabitants in 2015 [34]. This data was then combined with the data from the national drug database to create an age-matched population in order to calculate the proportion of inhabitants using statins in the county of Västerbotten. The Swedish National Diabetes Register (NDR) provides data on Swedish diabetes patients. This data can also be subdivided into counties such as Västerbotten [35]. Information on all patients diagnosed with DM II in Västerbotten in 2015 was retrieved from the NDR including use of blood lipid-lowering drugs. These data from the NDR were subtracted from the data from the population register and national drug database. This provided us with information on how many individuals were prescribed statins in Västerbotten, excluding DM II patients. This data was then age-matched to the study population. This was done by firstly dividing the groups into age strata (20–49, 50–54, 55–60, 65–69, 70–74, 75–79, 80–84, 85+ years of age). Then the proportion of individuals prescribed statins in Västerbotten was multiplied with the amount of individuals in each age stratum of the study group. By this method, we got an age-matched count of statin users in the study group possible to compare to the proportion in Västerbotten county.

Regarding the secondary endpoint, ‘recent’ statin users (including DM patients) were compared with the ‘former’ and ‘never’ users grouped together for analysis investigating whether or not there was a difference in cancer-specific survival.

### Statistical analyses

Statistical analyses were performed using STATA® version 14 (StataCorp LP, College Station, Texas, USA). The main comparison between the proportion of patients using statins in the study population and the proportion using statins in the population of Västerbotten (excluding individuals with DM type II) was calculated using a one-proportional one-sided *z* test.

‘Recent’ statin users in the study population were compared with ‘former’ and ‘never’ users together, and statistical analyses were performed on all variables collected from the medical record database. The *T* test was used for normally distributed continuous variables (age, BMI, haemoglobin, blood pressure), and the non-parametric Wilcoxon Mann-Whitney *U* test was used for continuous variables not normally distributed (CEA, CRP). The Wilcoxon Mann-Whitney *U* test was also used for ordinal variables (BMI, TNM, stadium) while chi-square was used for binary and categorical variables.

The secondary outcome survival analysis was visualised with Kaplan-Meyer curves calculated with log-rank test. A Cox regression hazard was also conducted, both by uni- and multivariate models. All variables were tested for proportional assumption. The endpoint used in survival analysis was cancer-specific survival, calculated by data retrieved from death certificates when available.

### Ethical approval

Ethical approval for this study was processed and approved by the Regional Ethics Review Board in Umeå, reference number 2016/201-31.

## Results

**Fig. 1:**
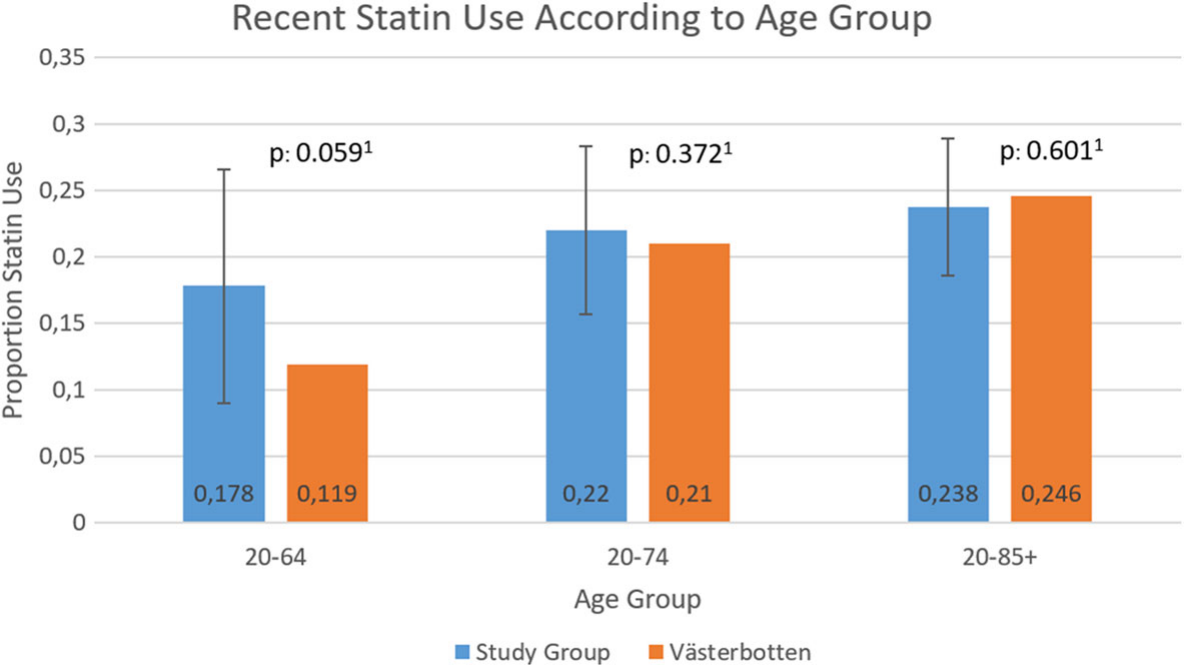
‘Recent’ statin users in the study population compared with the background population in the county of Västerbotten. *p* values calculated with one-proportional one-sided *z* test and error bars representing 95% confidence interval

With DM II patients excluded, 260 consecutive cases of colorectal cancer diagnosed at NUS between 2012 and 2015 were identified. Characteristics of the study population, including subgroup data for patients aged 20–64 years, are summarised in Table 1. The mean age at diagnosis was 70.4 years, and 51.4% of the patients were male. The majority of statin users were recent and long-term users. The most commonly prescribed statin was simvastatin. 28.1% of the study population were ‘has used’ users of statins. Since statin use in our control group was defined as statins prescribed during the last year, we used ‘recent’ users in the calculations. 23.8% of the patients in our study group were ‘recent’ users of statins while the corresponding age-matched figure in Västerbotten was 24.6%, a non-significant difference (23.8%, 95% CI 18.6–29.0%, *p* = 0.601) Fig. 1. When considering study and control groups 20 to 64 years of age only, the difference was slightly greater but did not reach significance, 17.8 versus 11.9%, respectively (17.8%, 95% CI 9.0–26.6%, *p* = 0.059).

**Table 1:** Characteristics of the study population (*n* = 260, diabetic patients excluded)

Characteristic	Cases (*n* = 260)	Age group 20–64 years old (*n =* 73)
Age (year)	70.4 (SD 12.7)	–
Male gender (%)	51.2	54.8
Use of statins (%)		
Never	71.9	80.8
Has used	28.0	19.2
Recent (% of ever)	84.9	92.9
Former (% of ever)	15.1	7.1
Length of statin use (%)		
Short term	27.4	21.4
Medium term	13.7	28.6
Long term	58.9	50.0
Type of statin use (%)		
Simvastatin	52.1	71.4
Atorvastatin	8.2	7.1
Mix of statins	39.7	21.4
Diagnosis (%)		
Rectal cancer, C20	38.9	54.8
Colon cancer, C18	61.1	45.2
Use of NSAIDs (%)		
Never/rare	56.2	60.3
Has used	43.9	39.7
Recent (% of ever)	40.4	44.8
Former (% of ever)	59.7	55.2
Use of salicylic acid (%)		
Never/rare	76.9	91.8
Has used	23.1	8.2
Recent (% of ever)	83.3	83.3
Former (% of ever)	16.7	16.7
BMI (kg/m^2^)		
Mean	25.7 (SD 4.5)	26.7 (SD 4.5)
Median	25.1	26.3
IQR	5.8	7.0
Smoking (%)		
Never	23.4	16.2
Currently	9.1	13.2
Prior	42.4	27.9
Not currently	25.1	42.6
Atherosclerosis (%)		
Yes	15.0	4.1
Myocardial infarction (%)		
Yes	8.1	2.7
Stroke (%)		
Yes	8.1	1.4
Alcoholism (%)		
Yes	3.5	6.8
Cholecystectomy (%)		
Yes	8.9	2.7
CRP at diagnosis (mg/mL)		
Mean	24.3 (SD 48.9)	19.4 (SD 32.5)
Median	5.5	5.5
IQR	21.8	15.4
CRP > 10 mg/mL (%)		
Yes	37.3	30.9
Hb at diagnosis (g/L)		
Mean	123 (SD 22.6)	127 (SD 22.6)
Median	126	132
IQR	29.3	34
CEA at diagnosis (ng/mL)		
Mean	38.4	23.3
Median	3.5	3.0
IQR	11.9	12.0
CEA > 5 ng/mL (%)		
Yes	41.0	36.2
Tumour, TNM (%)		
TX	5.1	1.4
T1	5.1	5.7
T2	13.7	10.0
T3	53.5	60.0
T4	22.7	22.9
Nodes, TNM (%)		
NX	8.2	–
N0	45.9	43.7
N1	22.2	25.4
N2	23.7	31.0
Metastasis, TNM (%)		
M0	79.6	76.7
M1	20.4	23.3
Stadium (%)		
I	15.0	12.7
II	30.1	26.8
III	33.3	36.6
IV	21.5	23.9
Previous cancer diagnosis (%)		
Yes	10.4	2.7
Heredity (%)		
No/unknown	89.2	86.3
First-grade relative	10.0	12.3
Hereditary nonpolyposis colorectal cancer	0.8	1.7
Familial adenomatous polyposis	–	–
Hereditary CRC	–	–
Colitis (%)		
No	97.7	95.9
Ulcerative colitis	0.8	1.4
Crohn	0.8	1.4
Ischaemic	0.4	–
Other	0.4	1.4
Systolic BP (mmHg)		
Mean	137 (SD 20.5)	135 (SD 17.1)
Median	135.5	133.5
IQR	28.3	26.5
Diastolic BP (mmHg)		
Mean	79 (SD 11.8)	83 (SD 10.4)
Median	80	85
IQR	18.8	15

*NSAID* non-steroidal anti-inflammatory drug, *BMI* body mass index, *CRP* C-reactive protein, *Hb* haemoglobin, *CEA* carcinoembryonic antigen, *BP* blood pressure, *IQR* inter-quartile range, *SD* standard deviation

For analysis of the secondary endpoint, difference in survival between statin ‘has used’ and ‘former/never’ users, the entire study population was used, including patients diagnosed with DM II. Comparison of patient characteristics between ‘recent’ statin users and ‘former/never’ statin users is shown in Table 2 with corresponding *p* values. The ‘recent’ statin users were significantly older than ‘former/never’ statin users. Furthermore, ‘recent’ statin users were more likely to have diabetes, stroke, myocardial infarction and atherosclerotic disease and had a significantly higher BMI and lower diastolic blood pressure at diagnosis.

**Table 2:** Comparison between statin ‘recent’ users and ‘former/never’ used

Characteristic	Statin recent users (*n* = 107)	Statin former/never used (*n* = 218)	*p* value
Age (year)	73.9 (SD 9.49)	70.1 (SD 13.2)	0.0075^1^
Male gender (%)	55.1	51.4	0.523^2^
Diabetes			< 0.0001^2^
Yes (%)	43.0	9.2	
Diagnosis (%)			0.440^2^
Rectal cancer, C20	41.1	36.7	
Colon cancer, C18	58.9	63.3	
Smoking (%)			0.175^2^
Never	19.4	23.4	
Not currently	18.3	23.4	
Currently	11.8	8.6	
Prior	50.5	39.9	
No info	*N* = 14	*N* = 20	
Current smoker (%)			0.382^2^
Yes	11.8	8.6	
Use of NSAIDs (%)			0.743^2^
Has used	44.9	46.8	
Never/rare	55.1	53.2	
Use of salicylic acid (%)			< 0.0001^2^
Never/rare	38.3	85.8	
Has used	61.7	14.2	
BMI, kg/m^2^			0.0002^1^
Mean	27.8	25.6	
Median	27.4	24.9	
IQR	7.0	6.0	
BMI (kg/m^2^) (%)			0.0001^3^
< 24.9	31.1	50.2	
25–30	35.8	34.4	
> 30	33.0	15.3	
CRP at diagnosis, mg/L			0.887^3^
Mean	24.1	23.2	
Median	6.0	6.0	
IQR	22.1	20.9	
CRP > 10 mg/L (%)			0.991^2^
Yes	37.6	37.6	
Hb at diagnosis, g/L			0.153^1^
Mean	119	123	
Median	122	126	
IQR	32	31	
CEA at diagnosis, ng/mL			0.777^3^
Mean	154	29.5	
Median	3.5	3.7	
IQR	18.8	10.3	
CEA > 5 ng/mL (%)			0.270^2^
Yes	35.8	42.6	
Tumour, TNM (%)			0.499^3a^
TX	8.6	4.2	
T1	2.9	4.7	
T2	15.2	13.5	
T3	55.2	54.0	
T4	18.1	23.7	
Nodes, TNM (%)			0.882^3b^
NX	11.3	7.9	
N0	45.3	45.8	
N1	19.8	21.8	
N2	23.6	24.5	
Metastasis, TNM (%)			0.448^2^
M0	76.6	80.3	
M1	23.4	19.7	
Stadium (%)			0.720^3^
I	14.6	14.1	
II	29.1	30.7	
III	32.0	34.1	
IV	24.3	21.0	
Previous cancer diagnosis (%)			0.673^2^
Yes	13.1	11.5	
Heredity (%)			0.446^2^
No/unknown	92.5	89.0	
First-grade relative	7.5	10.1	
Hereditary nonpolyposis CRC	–	0.9	
Familial adenomatous polyposis	–	–	
Hereditary CRC	–	–	
Colitis (%)			0.437^2^
No	98.1	98.2	
Ulcerative colitis	–	0.9	
Crohn	0.9	0.5	
Ischaemic	–	0.5	
Other	0.9	–	
Systolic BP, mmHg			0.469^1^
Mean	139	137	
Median	140	137	
IQR	20.5	30	
Diastolic BP, mmHg			0.028^1^
Mean	77	80	
Median	77.5	80	
IQR	15	18	
Cholecystectomy (%)			0.821^2^
Yes	8.4	9.2	
Atherosclerosis (%)			< 0.001^2^
Yes	43.9	6.4	
Myocardial infarction (%)			< 0.001^2^
Yes	21.5	3.7	
Stroke (%)			< 0.001^2^
Yes	20.6	4.6	
Alcoholism (%)			0.488^2^
Yes	1.9	3.2	

Characteristics of the study population, total number of observations 325 (including DM II). Comparison between ‘recent’ statin users versus never and former statin users. Statistical calculations indicating which variables differ between the two groups

*NSAID* non-steroidal anti-inflammatory drug, *BMI* body mass index, *CRP* C-reactive protein, *Hb* haemoglobin, *CEA* carcinoembryonic antigen, *BP* blood pressure, *IQR* inter-quartile range, *SD* standard deviation, ^1^ *t* test,^2^ chi-squared, ^3^ Wilcoxon Mann-Whitney

^a^Only calculated on T1, T2, T3 and T4

^b^Only calculated on N0, N1 and N2

**Fig. 2:**
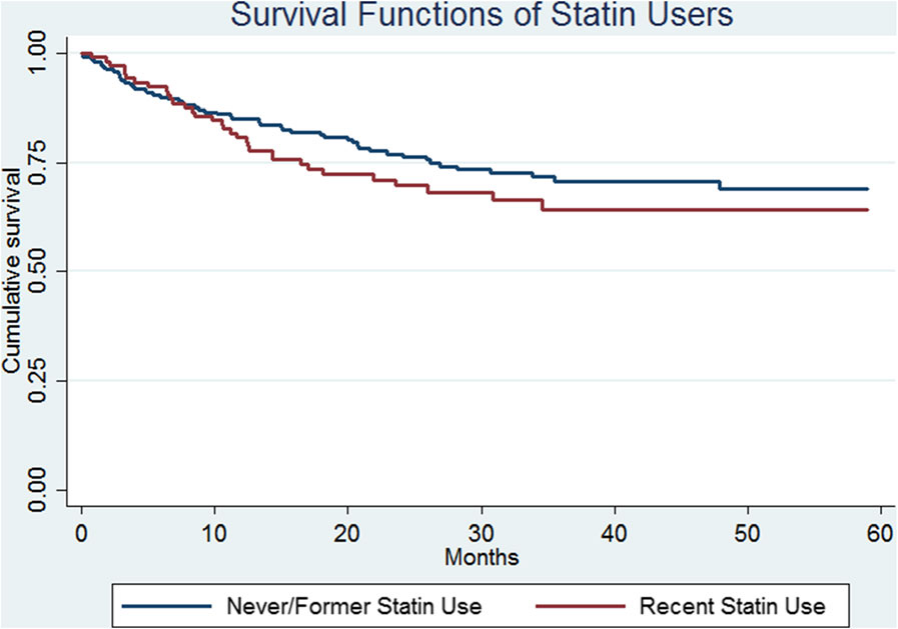
Kaplan-Meier curve of survival function for ‘never/former’ statin users versus ‘recent’ users. The endpoint was set to cancer-specific survival. Calculations for the curve were made with log-rank test and a *p* value of 0.15

Univariate Cox regression showed no significant difference in survival between ‘recent’ statin users and ‘former/never’ statin users (HR 1.39, 95% CI 0.89–2.16). Univariate Cox regression with statin use and a *p* value < 0.05 were entered into a multivariate Cox regression model as shown in Table 3. Age and cancer stage were the only significant variables in the multivariate model. A Kaplan-Meier curve showing cumulative survival of ‘recent’ statin users and ‘former/never’ statin users was created (Fig. 2). This showed a non-significant difference in survival using the log-rank test (*p* value 0.15).

**Table 3:** Cox regression for cancer-specific survival

Cancer-specific survival	Univariate analysis HR (95% CI)	Multivariate analysis HR (95% CI)
Statin use		
‛Former/never’	Ref 1.0	Ref 1.0
‛Recent’	1.39 (0.89–2.16)	1.12 (0.64–1.96)
Age		
< 70	Ref 1.0	Ref 1.0
70–80	1.79 (1.04–3.07)	1.89 (1.09–3.27)
> 80	2.76 (1.61–4.75	2.67 (1.49–4.76)
Gender		
Male	Ref 1.0	
Female	1.11 (0.72–1.72)	
Diagnosis		
Colon	Ref 1.0	
Rectum	0.78 (0.49–1.24)	
Diabetes		
No	Ref 1.0	
Yes	1.56 (0.95–2.55)	
Previous cancer diagnosis		
No	Ref 1.0	
Yes	1.24 (0.66–2.34)	
Stage		
I + II	0.31 (0.14–0.68)	0.33 (0.15–0.73)
III	Ref 1.0	Ref 1.0
IV	7.63 (4.47–13.0)	8.70 (8.70–15.1)
BMI		
< 24.9	Ref 1.0	
25–29.9	0.69 (0.42–1.15)	
> 30	0.91 (0.52–1.59)	
NSAID use		
Former and never	Ref 1.0	
Recent	0.57 (0.28–1.14)	
Salicylic acid use		
Former and never	Ref 1.0	Ref 1.0
Recent	1.67 (1.04–2.68)	1.39 (0.75–2.60)

Cox regression both univariate and multivariate model with cancer-specific survival as endpoint

*HR* hazard ratio, *CI* confidence interval, *Ref* reference, *BMI* body mass index, *NSAID* non-steroidal anti-inflammatory drug

## Discussion

In this pilot study, we found no correlation between statin use and CRC. This is in accordance with previous meta-analyses in the field [16, 36], even though results from previous studies diverge. Almost all meta-analyses on CRC and cancer development performed after 2006 include a study by Poynter et al. [21] showing a 47% risk reduction in the development of CRC in a population prescribed with statins compared to a control group. This study has been criticised for not having considered several confounding factors [37]. For this reason, meta-analyses on this subject should be interpreted with caution.

It is well known that statins reduce the risk for cardiovascular events and recent studies support the use of statins as primary prevention [38, 39]. Swedish guidelines recommend that patients with a 10-year risk of death from a cardiovascular event higher than 5% should receive low-dose statin treatment (simvastatin 20–40 mg per day) regardless of lipid levels [40]. This is a modification of previous recommendations where the same patient group was recommended changes in lifestyle rather than prescription of a statin [41]. This modification follows American guidelines for primary prevention of cardiovascular disease [42] but not those in Europe [43]. When comparing the study and background populations aged 20–64 years, we found a slightly higher risk in patients prescribed statins, but this difference was non-significant. This is an age group where the new recommendations on statins have a great impact due to the scoring diagram (SCORE) that is widely used in Sweden [44]. This is also an age group with a long life expectancy, and if they are prescribed statins before the age of 65, they are likely to be lifelong users.

A secondary aim of this study was to see if there was any difference in survival between ‘recent’ and ‘former/never’ users of statins in patients diagnosed with CRC. In this endpoint, patients diagnosed with DM II was included since diabetes is an established risk factor for the development of CRC [33] but does not affect cancer-specific survival [45]. In this study, no significantly difference was seen. Kaplan-Meier survival curves also revealed no difference. This is in accordance with a recently published cohort study including 41,900 patients [46]. Since statins are immune-modulating [47], it is possible that symptoms are delayed and that patients using statins receive their diagnosis at a later stage and therefore have a lower survival rate. In the multivariate Cox regression model, the only variables with a significant impact on the hazard ratio were increasing age and stage, giving a higher hazard ratio, i.e. higher mortality correlates with older age and more extensive disease.

It is not surprising that more ‘recent’ statin users had a history of myocardial infarction, stroke, diabetes and atherosclerotic disease as well as higher BMI than the ‘never/former’ statin users. This is important to remember when interpreting the results of the cancer-specific analysis since myocardial infarction and stroke increase mortality risk. We therefore used cancer-specific survival as endpoint in this study. This skewness of groups is also important when interpreting the results of analysis of the primary endpoint in this study since high BMI and diabetes both are risk factors for the development of CRC [33, 48] as well as myocardial infarction and stroke.

### Limitations

For a matched-control study, observations for each variable are required. Since data on statin use in Västerbotten were collected as a proportional number, we did not have observations for each variable. Since our control group was matched for age only, it was impossible to adjust for confounders such as previous radiation in the area, BMI and smoking habit. The only risk factor adjusted for was DM type II since this is a risk factor both for CRC and statin prescription. It is not possible to say whether or not statin users are more likely to have a higher BMI or to be more frequent smokers than those who have never used statin. Should that be the case, this would have led to a higher risk regardless of statin use per se. In our secondary endpoint, the survival analysis, we compared the statin users with the non-statin users among patients diagnosed with CRC. Thus, observations were available for each patient in the analysis and multivariate analysis was performed.

The statin prescription data from the medical records are data for prescriptions. These were compared with the National drug database, which provides information from the pharmacists, and those data are retrieved drugs at the pharmacy. Prescribed data are probably overestimated. However, to adjust for this, statin use was defined as ‘two or more statin prescriptions’, as has been done in previous studies in this field [30].

## Conclusion

We found no significant increase in risk for developing colorectal cancer in patients medicated with statins (diabetic patients excluded). We found no correlation between statin use at diagnosis and cancer-specific survival.

## Abbreviations

BMI: Body mass index
CRC: Colorectal cancer
DM II: Diabetes mellitus type II
MtDNA: Mitochondrial DNA
ROS: Reactive oxygen species nDNA––nuclear DNA
TNM: Tumour, node, metastasis
